# Primary Sleeve Gastrectomy and Leaks: The Impact of Fundus-Wall Thickness and Staple Heights on Leakage—An Observational Study of 500 Patients

**DOI:** 10.3389/fsurg.2021.747171

**Published:** 2021-10-21

**Authors:** Clara Boeker, Barbara Schneider, Valentin Markov, Julian Mall, Christian Reetz, Ludwig Wilkens, Ibrahim Hakami, Christine Stroh, Hinrich Köhler

**Affiliations:** ^1^Department of General, Visceral, Vascular and Bariatric Surgery, Klinikum Nordstadt, Hannover, Germany; ^2^Department of Pathology, Klinikum Nordstadt, Hannover, Germany; ^3^Department of Psychology, University of Hildesheim, Hildesheim, Germany; ^4^Department of General, Visceral and Bariatric Surgery, College of Medicine at Jazan University, Jizan, Saudi Arabia; ^5^Department of Bariatric Surgery, Stiftung Rehabilitation Heidelberg Waldklinikum, Gera, Germany; ^6^Department of General, Visceral and Bariatric Surgery, Herzogin Elisabeth Hospital, Braunschweig, Germany

**Keywords:** fundus-wall thickness, leak, sleeve gastrectomy, staple heights, bariatric, surgery

## Abstract

**Introduction:** The most feared complication of laparoscopic sleeve gastrectomy (LSG) is staple-line leakage. Staple height and fundus-wall thickness might influence such leakage, and this study examined their possible impact on leak incidence. Factors including gender, age, comorbidities, and reinforcement of the staple line were also investigated.

**Methods:** A total of 500 patients between 17 and 71 years of age who were scheduled for LSG were selected to participate in the study. For technical reasons, 53 were excluded. The fundus-wall thickness of 447 patients after LSG was investigated. The impact of staple height, fundus-wall thickness, demographic and medical factors on leak incidence were investigated. Most of our patients (309) were female (69%), while 138 were male (31%).

**Results:** The mean thickness of the proximal fundus wall was 2,904 μm, 3,172 μm in men and 2,784 μm in women. The leak rate was 4.9%. Age, fundus-wall thickness, and BMI showed a strong influence on leak risk, but this effect was significant only for age (*p* = 0.01). Patient gender and staple size showed no significant influence on the correlation between fundus-wall thickness and leak risk. Gender displayed a small effect of influence on this correlation, with η*2* = 0.05.

**Discussion:** Because older age had a significant effect on increasing the risk of staple-line leakage, there is a need for a more specific focus on these patients. Thinner fundus wall and female gender might predispose patients to staple-line leaks, but a significant value could not be reached. Therefore, staple size should remain the surgeon's choice based on clinical experience.

## Introduction

Severe obesity is a life-threatening disease, one that is known to cause premature death (1). Bariatric surgery is the most effective therapy available to the obese, and of the available procedures, laparoscopic sleeve gastrectomy (LSG) is among those most frequently performed worldwide (2, 3). LSG results in long-term reduction of weight and its co-morbidities comparable to the benefits of the roux-en-y gastric bypass (RYGB) (4). Yet these drastic weight-loss measures are not without risk. One of the most feared complications of bariatric surgery is staple-line leakage, which can end in sepsis, peritonitis, and even death (5). The underlying causes of such complications remain unclear. In addition to demographic and medical aspects, such as age, sex, and BMI (5), technical factors including thermal injury, reinforcement of the staple line, or bougie size were shown to predispose patients for staple-line leakage (6–11). Staple height and fundus-wall thickness have also been discussed as parameters that may increase the risk of developing a leak (6, 12–14). The fundus wall is demonstrably thinner than the wall of the corpus and antrum, and is therefore at greater risk of becoming a leakage site (12). In addition, staple height must correlate properly with the dissected fundus wall so as to correctly adapt the tissue to achieve hemostasis while avoiding ischemia of the tissue (8).

In an earlier study, we investigated the correlation between fundus-wall thickness and clinical and demographic data. A correlation was seen only for gender, with a thicker fundus wall in male patients. During the review process for that paper, the reviewers noted the small sample size (141 patients), and stated that it was insufficient to show any significant influence of staple-line leakage in a study population with a leak likelihood as small as 2%. Thus, no further correlation could be found within this group of patients, only three of whom suffered leakage (15). For this reason, we continued our measurements and investigated another 306 gastric specimens. The results found for this cohort of 447 patients are presented here. Surgical technique was changed during the study period: The staple line was reinforced by either Gore Seamguard® or oversuture, while bougie size was changed from 34 to 40 Charrier.

The aim of our recent work was to determine the impact of fundus-wall thickness and staple heights on leak risk after LSG. Factors such as gender, age, comorbidities, and reinforcement of the staple line and their influence on leak likelihood were also investigated.

## Materials and Methods

### Study Design and Patient Acquisition

This investigation was part of an observational study. Initial data were published in 2017 (15). The study was approved by the local ethical committee (Hannover Medical School, Hanover, Germany. No. 2760-2015). The principles of the Declaration of Helsinki for biomedical research were followed. All procedures met the criteria of the German guidelines for the treatment of obesity and bariatric surgery (16–18). There were no exclusion criteria.

Between January 2014 and January 2021, 500 patients between 17 and 71 years of age who were scheduled for primary LSG gave their informed consent to participate in the study. Because of major lesion of the proximal fundus wall during extraction of the specimen from the abdominal cavity, or due to torsion of the specimen during the fixation process, both of which impaired an exact measurement of the vertical gastric wall, 53 specimens had to be excluded from further assessment. The fundus-wall thickness of 447 patients following LSG was investigated. A correlation of leak incidence with fundus-wall thickness, staple size, demographic and medical data was analyzed. Most of our patients were female (309 or 69%) vs. 138 who were male (31%). Patients' characteristics are listed in Table 1.

**Table 1:** Patient characteristics.

Gender	Male	138	30.9%
Gender	Female	309	69.1%
BMI	≤ 50	189	42.3%
BMI	>50	258	57.7%
Comorbidities	Hypertension	224	50.1%
Comorbidities	Diabetes mellitus	105	23.5%
Mean age	42		

*BMI: kg/m^2^*.

### Surgical Technique

In the first 141 procedures, gastric sleeves were dissected over a 34-Charrier bougie, while the remaining 306 specimen were dissected over a 40-Charrier bougie. The greater curvature was dissected using the Endo-Cutter Echelon^TM^ Flex60 from Ethicon Endo-Surgery®, starting with 4–6 cm proximal of the pylorus using two green cartridges (open staple height, 4.1 mm; closed staple height, 2.0 mm), followed by gold cartridges with smaller staple height (open staple height, 3.8 mm; closed staple height, 1.8 mm). The fundus was resected with either gold or blue cartridges (open staple height 3.5 mm, closed staple height 1.5 mm) toward the angle of His, according to the surgeon's preference. In the first 137 procedures, the staple line was not reinforced. In the remaining 310 procedures, the proximal staple line was reinforced by oversuture in 252 operations or buttressing using Seamguard in 43 operations as surgeon's preference. A drainage was placed next to the staple line to indicate leakage or bleeding. At post-operative day 2, each patient underwent a blue dye-swallowing test to exclude an acute leak, and then the drain was removed. As is routine, patients were dismissed at postoperative day 3 after a dietary consultation. When leakage was suspected, a CT scan and gastroscopy were performed. If leakage was discovered, a stent was implanted in the sleeve. Any abdominal abscesses were washed out and drained by re-laparoscopy.

### Follow-Up

As part of the follow-up regimen, our Bariatric Surgery Department routinely schedules all patients for postoperative consultations in the Outpatient Department at 2, 6, 12, 18, and 24 months following surgery. Further follow-up visits are scheduled once yearly.

### Specimen/Measurements

**Figure 1:**
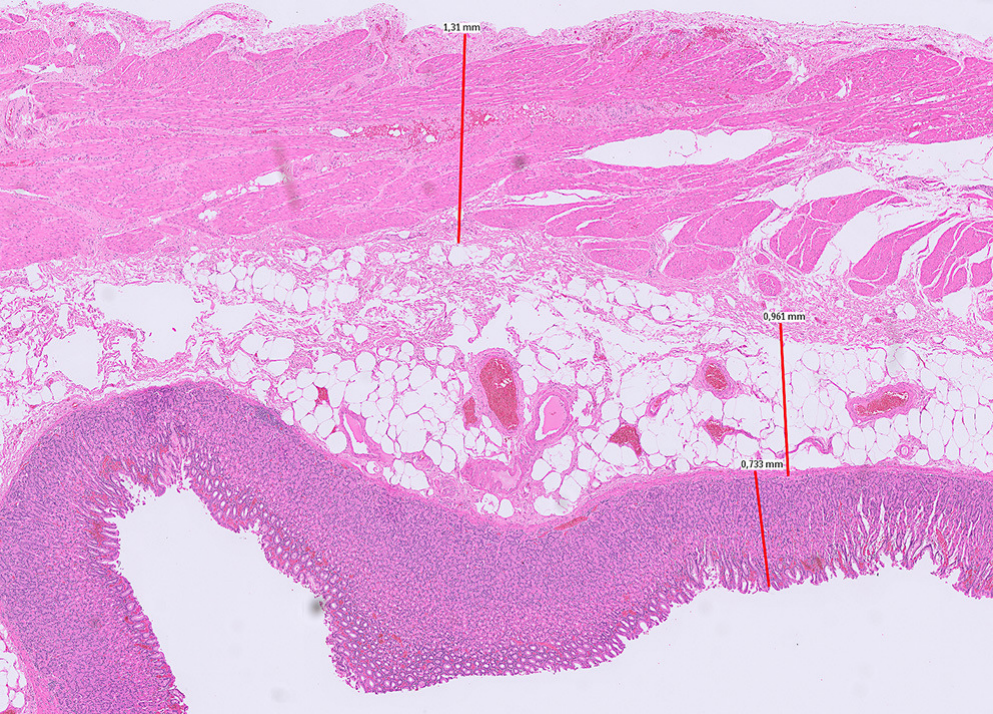
Measurement of fundus-wall thickness (mucosa, submucosa, muscularis propria). Light microscopy of fundus wall 1 cm down the proximal end of the staple line.

Throughout the study period, the preparation, staining, and analysis of specimens for microscopic measurement did not change. Immediately after extraction of the specimen from the abdomen, the sampling point of the specimens was marked with a suture 1 cm down the proximal end of the staple line, fixed in formalin, and sent to the pathologist. Samples of the resected specimens were excised by the pathologist at the marked site and to the HE stained according to standard protocol. Measurements were performed via light microscopy. Mucosa, submucosa, and muscularis were measured separately at thinnest onslide point and summated (Figure 1).

### Statistical Analysis

Data analyses were performed using the IBM Statistical Package for Social Sciences (IBM SPSS, version 26). Impact of the nominal variables gender, staple size, high blood pressure, diabetes mellitus, comorbidity, Seamguard, and oversuture on leakage was calculated with a chi-squared test using Cramér's *V* as a statistical coefficient to measure the strength of association.

Relationship and significance between leak risk and the numeric variables age, fundus-wall thickness parameters, and BMI was computed by one-way analysis of variance (ANOVA) between subjects, with eta-squared coefficient for effect size (η*2*) and *p*-value for significance. The influence of the variables gender and staple size on the relationship between fundus-wall thickness and leak risk was calculated using analysis of covariance (ANCOVA) (19–21).

## Results

The mean thickness of the proximal fundus wall was 2,904 μm, 3,172 μm in men and 2,784 μm in women. The mean values of the different layers (mucosa, tela submucosa, muscularis propria) are shown in Table 2. Mean BMI was higher in men.

**Table 2:** Descriptive data of mean fundus-wall thickness, BMI, and gender.

	**Musc. prop**		**submucosa**		**Mucosa**		**Fund. wall**		**BMI**		**n**
	**M**	**SD**	**M**	**SD**	**M**	**SD**	**M**	**SD**	**M**	**SD**	
male	1039	550	1052	592	1080	324	3172	1098	53	9	138
female	899	470	889	505	996	302	2784	965	51	9	309
all	942	500	939	538	1022	311	2904	1022	52	9	447

*Mean values of wall thickness in μm, BMI in m^2^/kg*.

### Chi-Squared Test

All in all, 22 patients experienced post-operative leaks, which accounted for an overall leak frequency of 4.9% (2.2% in males and 6.6% in females). The calculation of chi-square analysis revealed no effects and no significant correlation between leakage and six nominal variables: staple size, high blood pressure, diabetes mellitus, comorbidity, Seamguard, and oversuture. Only the variable gender showed a small effect (Cramér's *V* = 0.10) and a trend of significance (*p* = 0.07) on leak incidence. This points to a possible higher risk for females. The fourfold correlation between leakage and gender is shown in Table 3.

**Table 3:** Correlation of leakage and gender.

	**Female**	**Male**	**All**
No leaks	290	135	425
Leaks	19	3	22
All	309	138	447

*Cramér's V-value = 0.09, small effect; p = 0.071 (p > 0.05)*.

Cramér's *V* values are conventionally interpreted as a small effect for *V* = 0.1, a medium effect for *V* = 0.3, and a strong effect up to *V* = 0.5 (19).

The values of the six non-significant correlations are referenced as follows: In 228 cases, the staple size used for the last firing at the angle of His was a gold cartridge, and in 219 cases it was a blue cartridge. With 11 leaks in both groups, there was no significance detectable for staple size (Cramér's *V* = 0.01, sign. *p* = 0.90), nor did analysis of the correlation between leak occurrence and comorbidities reveal an effect (Cramér's *V* = 0.04,), sign. *p* = 0.45 (*p* > 0.05), especially in the case of high blood pressure (Cramér's *V* = 0.02,), sign. *p* = 0.67 (*p* > 0.05) and diabetes mellitus (Cramér's *V* = 0.03), sign. *p* = 0.54 (*p* > 0.05). Furthermore, there was no correlation between leakage and the use of Seamguard (Cramér's *V* = 0.03), sign. *p* = 0.52 (*p* > 0.05) or oversuture of the proximal staple line (Cramér's *V* = 0.05), sign. *p* = 0.22 (*p* > 0.05).

### Anova

One-way ANOVA showed strong effects of influence for age, fundus-wall thickness, and BMI on leak risk. This strong effect was only significant for age (Table 4); in other words, the older the patient, the higher the risk for developing a leak. The correlation of BMI and leak risk revealed a strong effect, with η = 0.71, or η*2* = 0.50, but no significance. No trend of higher leak risk in higher-body-weight patients was detectable. The mean BMI of patients without leakage was 51.9 m^2^/kg (n = 423), while the mean BMI for patients who experienced a leak was 49.8 m^2^/kg (n = 22); η*2* < 0.06, small effect; η*2* = 0.06–0.14, medium effect; η*2* > 0.14, strong effect (19).

**Table 4:** Correlation of metric variables and leakage.

**Metric variables**	**η**	***η2***	***p***
Age	0.42	0.17	**0.01**
Muscularis propria	0.82	0.67	1.00
Tela submucosa	0.92	0.85	0.45
Mucosa	0.85	0.72	0.36
Entire fundus wall	0.93	0.87	0.97
BMI	0.71	0.50	0.57

*η and η2 = effect size coefficients; p = value of significance; p > 0.05 = non-significant; η2 <0.06, small effect; η2 = 0.06–0.14, middle effect; η2 > 0.14, strong effect. Bold values are significance*.

With respect to leaks and fundus-wall thickness, the one-way ANOVA revealed the strongest effect for the thickness of the tunica muscularis propria. The same trend was seen for the tunica mucosa, tela submucosa, and the entire fundus wall. In each category, the wall thickness was slighter in the leak group, but according to these effects, significant values could not be reached, meaning that a thinner fundus wall may be more likely to develop a leak (Table 5). The strong effect is due to large differences between groups of leak patients and non-leak patients, but might also be caused by chance, seeing as significant values could not be reached.

**Table 5:** Descriptive data of age, fundus-wall thickness, and BMI.

	**Leaks**	**N**	**M**	**SD**	**SE**
Age	0	425	40.91	11.414	0.555
	1	22	42.64	12.730	2.714
Muscularis propria	0	425	951.09	502.816	24.448
	1	22	799.68	434.548	92.646
Submucosa	0	425	946.65	544.734	26.486
	1	22	833.27	390.550	83.265
Mucosa	0	425	1026.12	313.494	15.243
	1	22	963.55	268.629	57.272
Entire fundus-wall thickness	0	425	2923.85	1030.244	50.092
	1	22	2596.50	840.104	179.111
BMI	0	425	51.9482	8.94129	0.43474
	1	22	49.8818	7.16444	1.52746

*M = mean value, SD = standard deviation, SE = standard error, wall thickness in μm*.

### Ancova

An analysis of covariance (ANCOVA) with gender and staple size (blue or gold cartridge) as covariate factors showed no significant influence on the correlation between the thickness of the entire fundus wall and the incidence of leakage. Gender as a covariate variable displayed a small effect, with η = 0.22, η2 = 0.05, *p* = 0.20. For staple size as a covariate variable, no effect was seen: η = 0.00, η*2* = 0.00, *p* = 1.00.

## Discussion

In our sample, greater age was significantly associated with a higher incidence of staple-line leakage. This issue has become controversial in the literature; interestingly, Benedix et al. reported more leaks in younger patients in 2014 (5). In a later-reported larger cohort, there was no difference in mean age for patients with a leak vs. patients without a leak (22). The same results were reported by Sakran for a large Israeli cohort (23). Another study conducted by Benedix et al. in 2017 comparing the leak likelihood of adolescents to that of adults revealed no difference (1.9 vs. 1.4%) (24).

Our investigation of 447 LSG specimen indicated that fundus-wall thickness influenced the development of leaks. Staple-line leaks after LSG occur close to the angle of His in 90% of patients (25); for this reason, we analyzed the wall thickness of this area. There was a strong effect for the tunica muscularis propria on leakage. The same trend was seen for the tunica mucosa propria, the tela submucosa, and the whole fundus wall. In each category, the fundus wall was thinner in the leak group, meaning that a thinner fundus wall may have influenced the development of leaks. However, statistical significance could not be reached.

Another finding of this study was a strong effect of gender and BMI on leak occurrence, although significance could not be reached. Fundus walls were thinner for female vs. male subjects, and there was a higher incidence of leaks in females (6.6%) than in males (2.2%). This might indicate a higher risk of leakage due to thinner fundus walls for female patients in our study group. But with little influence and no significance, gender as a covariate variable showed only a small effect on the likelihood of developing a leak.

These results are in line with previously published data that showed thicker fundus walls in male patients, among the 141 fundus-wall-thickness measurements performed by our group (15). Because three patients of the leak group were female, and male gender was associated with thicker tissue, we concluded that female patients might be at higher risk for developing a leak due to a thinner fundus wall. In contrast to this finding, Benedix et al. reported significantly more leaks among male patients in a large German cohort (5).

Elariny et al. were the first to show a decreasing gastric wall thickness from the antrum to the fundus, with a thicker fundus wall for male patients. The same authors stated that a thicker gastric wall in males might be due to thicker muscle tissue (12). Rawlins and Rawlins found that the antrum is thicker in male patients with a BMI above 50 kg/m^2^ (13). Lee et al. reported a thicker gastric wall in patients whose characteristics included advanced age, male sex, diabetes mellitus, and smoking (26), but the sample size was small (*n* = 30), and average BMI was below 40 kg/m^2^. Correlation of staple-line leakage was not investigated in these studies.

The presence of a thinner wall might lead to the conclusion that smaller staple sizes are to be used at the angle of His. Abu-Ghanem showed the safety of smaller staple heights as hemostasis is provided (27). Previously, other authors had questioned the safety of using smaller staple heights (6). Huang and Gagner postulated that a measuring device is needed to gauge the tissue intraoperatively and define the appropriate staple height (14). In the present investigation, staple size did not show a significant effect, nor did staple-line reinforcement or oversuture. Regarding an inconsistent study situation reported in the literature, staple size should remain the surgeon's choice based on experience (5, 28, 29).

Lower risk of developing a leak for patients with thicker fundus wall—as shown by our investigation—supports the findings of other studies, which revealed a vulnerable area in the proximal staple line. Marie et al. were able to demonstrate the fragility of the fundus wall under high pressure in the sleeve by investigating the bursting pressure and the probability of leakage (30).

Basso et al. discussed an ischemic region of the proximal sleeve in certain patients, depending on the existence and route of a posterior gastric artery (10). Also, surgical and tissue-based issues play a role in the occurrence of leaks; the incidence is multifactorial and entails local ischemia, increased intraluminal pressure, and extensive lateral traction during resection as well as viscosity of the tissue (31, 32).

To the best of our knowledge, this is the first study to investigate the fundus-wall thickness of a large patient cohort (447 in all) following LSG. The strength of our study may rest in the size of the study population; by comparison, former studies investigating fundus-wall thickness included no more than 60 patients. One limitation of this study might be the technique used for measurement, as the formalin-fixated tissue does not represent the *in vivo* situation; formalin fixation is known to cause tissue shrinkage of up to 20% (33). Nevertheless, all specimens had been prepared identically, such that a comparison within our study group was possible and reproducible. While the study population was large, the cohort of 447 patients was still too small to show statistical significance in all but one parameter we investigated, but it was possible to indicate trends.

Cartridge color (i.e., size) was the personal choice of the surgeon, and was not randomized. Additionally, strengthening the staple line by oversuture or buttressing in 295 of the last 306 procedures was not randomized.

Furthermore, we should discuss the high leak rate of 4.9% compared to what is reported in the current literature (3, 6, 22, 23, 25, 29, 34–36). This factor might result from a high BMI in our cohort (52 kg/m^2^), and from high turnover on our surgical team. We are a teaching hospital, and eight leaks occurred in the course of training junior surgeons. As a lesson learned, we reduced the team of operating surgeons to three senior bariatric surgeons, and adapted a stricter teaching curriculum. The surgical technique was changed to continuous oversuture of the staple line, and the resection of the fundus near the angle of HIS will be performed with 1cm distance from the esophagus, avoiding intense transversal traction of the fundus to the patient's left side.

In conclusion, the development of leaks following LSG is a multifactorial and complex process. While a thinner fundus wall along with female gender might predispose patients to staple-line leaks, significance could not be reached. Our study revealed that older age alone showed a significant negative influence on leak risk following LSG; cartridge size should remain the surgeon's choice based on clinical experience. With these things in mind, further research is needed to obtain more information about the impact of influencing factors on leakage following LSG.

## Data Availability Statement

The raw data supporting the conclusions of this article will be made available by the authors, without undue reservation.

## Ethics Statement

The studies involving human participants were reviewed and approved by Hannover Medical School, Hanover, Germany. No. 2760-2015. The patients/participants provided their written informed consent to participate in this study. Written informed consent was obtained from the individual(s) for the publication of any potentially identifiable images or data included in this article.

## Author Contributions

CB, JM, CR, IH, and HK contributed to conception and design of the study. CB and BS organized the database. BS and LW performed the histopathological measurements. VM performed the statistical analysis. CB and HK wrote the first draft of the manuscript. BS and VM wrote sections of the manuscript. CS reviewed the final article. All authors contributed to manuscript revision, read, and approved the submitted version.

## Conflict of Interest

The authors declare that the research was conducted in the absence of any commercial or financial relationships that could be construed as a potential conflict of interest.

## Publisher's Note

All claims expressed in this article are solely those of the authors and do not necessarily represent those of their affiliated organizations, or those of the publisher, the editors and the reviewers. Any product that may be evaluated in this article, or claim that may be made by its manufacturer, is not guaranteed or endorsed by the publisher.
